# Tolerability, safety and feasibility of metformin combined with chemoradiotherapy in patients with locally advanced cervical cancer: a phase II, randomized study

**DOI:** 10.2340/1651-226X.2025.43045

**Published:** 2025-03-19

**Authors:** Kjersti Skipar, Tord Hompland, Kjersti V. Lund, Christina S. Fjeldbo, Kristina Lindemann, Taran P. Hellebust, Heidi Lyng, Kjersti Bruheim

**Affiliations:** a Department of Oncology, Telemark Hospital Trust, Skien, Norway; b Department of Radiation Biology, Oslo University Hospital, Oslo, Norway; c Institute of Clinical Medicine, University of Oslo, Oslo, Norway; d Department of Radiology and Nuclear Medicine, Oslo University Hospital, Oslo, Norway; e Department of Surgical Oncology, Section for Gynecological Oncology, Oslo University Hospital, Oslo, Norway; f Department of Medical Physics, Oslo University Hospital, Oslo, Norway; g Department of Physics, University of Oslo, Oslo, Norway; h Department of Oncology, Oslo University Hospital, Oslo, Norway

**Keywords:** cervical cancer, multimodal imaging, hypoxia biomarkers, hypoxia modification, metformin, randomized phase II trial

## Abstract

**Background and purpose:**

Locally advanced cervical cancer is treated with chemoradiotherapy. The treatment-related morbidity is high. Tumor hypoxia has prognostic impact and represents a valid, interventional target. This phase II study investigated efficacy of the antidiabetic drug metformin to modify hypoxia according to established biomarkers. Preliminary results including tolerability, safety and feasibility are reported here.

**Patients and methods:**

Patients were included in a 1:1 randomized, open-label design, comparing standard chemoradiotherapy ± metformin. Metformin 850 mg twice daily was administered 1 week before and during chemoradiotherapy. Magnetic resonance images (MRI) and tumor biopsies were collected at baseline, after 1 week of metformin treatment, and at brachytherapy for biomarker assessments. Tolerability and safety were determined by treatment completion rates and frequency of adverse events (AEs). Safety was further evaluated by possible increase in MRI-based hypoxia during the first week of metformin. Feasibility was determined by proportion of completed study interventions and imaging and biopsy procedures.

**Results:**

In total, 18 and 23 patients were allocated to the intervention and control arm, respectively. Eighteen and 15 patients completed metformin treatment for 1 and 5 weeks. Frequency of AEs ≥ grade 3 was not significantly different between study arms. Most AEs were gastrointestinal toxicities. Tumors with increase in hypoxia during the first week were all below the defined safety limit. A total of 98% of scheduled MR series and biopsies were collected with satisfactory quality.

**Interpretation:**

Addition of metformin to chemoradiotherapy is tolerable and safe. Serial sampling of MRI and tumor biopsies for hypoxia biomarker assessment is feasible.

## Introduction

Patients with locally advanced cervical cancer (LACC) are treated with definitive chemoradiotherapy [1]. About one third of the patients experience disease recurrence, and further treatment improvements are warranted [1]. Currently, overall treatment-related morbidity is high with a significant impact on the patient quality of life [1]. The opportunity for further treatment intensifications might therefore be limited by patient tolerability. Hence, improved patient selection is important, as intensified treatments should be confined to patients at risk of treatment failure [1, 2].

Tumor hypoxia is an aggressive biological feature of LACC with established negative prognostic impact [3, 4]. Hypoxia therefore represents a valid target for treatment interventions that may improve patient outcome [5]. Several strategies to target hypoxia in LACC have been studied in clinical trials, and survival benefits have been reported [5]. However, beneficial strategies have not reached clinical practice, mostly due to logistical challenges and/or excessive toxicity. Further, most studies have been conducted before the introduction of concomitant chemotherapy [5], which impacts overall relevance. Clinically feasible and tolerable methods for hypoxia modification, with proven efficacy in LACC patients treated with chemoradiotherapy, are therefore warranted.

Over the last decade, metformin, which is a widely used antidiabetic drug, has been increasingly investigated for a role in cancer treatment [6, 7]. A possible value as a hypoxia modifying drug has been highlighted [8, 9]. Metformin inhibits complex 1 of the mitochondrial respiratory chain, which results in reduced cellular oxidative phosphorylation [8]. The subsequent reduction in cellular oxygen consumption has been shown to decrease tumor hypoxia in cervical cancer xenografts, by making more oxygen available in initially hypoxic tumor areas [10]. Moreover, metformin may improve tumor oxygenation by selectively killing hypoxic cells through the suppression of the mammalian target of rapamycin (mTOR) pathway and modulation of the unfolded protein response (UPR), both important for cell survival under hypoxic conditions [11, 12].

Metformin has a known safety profile in diabetic patients, and at antidiabetic dosage, the drug is well-tolerated with few serious side-effects [13]. Metformin combined with radiotherapy or chemoradiotherapy has been studied in patients with prostate cancer and locally advanced lung cancer, respectively, without any excess toxicities [14, 15]. Metformin has further been found to be tolerable in combination with chemoradiotherapy in LACC patients, although firm conclusions from this study could not be made due to a limited number of patients included [16]. Overall, metformin represents an exciting approach for hypoxia modification in LACC if efficacy is proven at tolerable doses.

Clinical benefits of hypoxia modifying drugs are expected to be limited to patients with hypoxic tumors, and hypoxia-targeting interventional trials should therefore include applicable methods for hypoxia assessment [17]. Such methods allow for upfront patient selection, and for documentation of drug effects by repeated measurements during the intervention [17]. We have previously developed hypoxia biomarkers based on magnetic resonance imaging (MRI) and gene expression levels in tumor biopsies [4, 18]. Imaging-based methods are appealing as they are non-invasive and provide information of the entire tumor, while alongside molecular analyses of tumor biopsies are important as they provide enhanced biological insight and validation [19]. Both of our biomarkers have been shown to provide prognostic information in independent LACC cohorts [4, 20], but prospective evaluation in a clinical interventional study is still lacking.

We have conducted a phase II, parallel, open label, randomized clinical study where metformin was combined with definitive chemoradiotherapy in LACC patients. The overall aim was to investigate whether metformin could reduce tumor hypoxia, according to our imaging- and gene-based biomarkers. A study design was applied where metformin was administered in the interventional arm 1 week prior to, and during chemoradiotherapy. MR images and tumor biopsies were collected at three consecutive time points, one before and two during therapy, to assess hypoxia and evaluate biomarker feasibility. No preselection of patients was performed, as a large heterogeneity in hypoxic fraction was considered beneficial for evaluation of our biomarkers. As a safety strategy, a possible increase in imaging-based hypoxia during the first study week was consecutively evaluated [21]. Preliminary results that include tolerability, safety and feasibility of the study intervention and methodology are reported her.

## Material and methods

### Patients and treatment

Patients were recruited from Oslo University Hospital between May 2020 and July 2024, and the last follow-up was in November 2024. Female patients ≥18 years of age with Eastern Cooperative Oncology Group (ECOG) performance status 0–1 and histologically confirmed cervical cancer FIGO_2018_ stage IB2-IVa, planned for curative chemoradiotherapy, were eligible for inclusion. Main exclusion criteria were evidence of distant metastases, other treatments for cervical cancer, known diabetes mellitus, currently on metformin or other antidiabetic drugs, uncontrolled intercurrent disease, conditions associated with increased risk of metformin-induced lactic acidosis and other invasive malignancies within last 12 months, with the exception of non-melanoma skin cancers.

**Figure 1:**
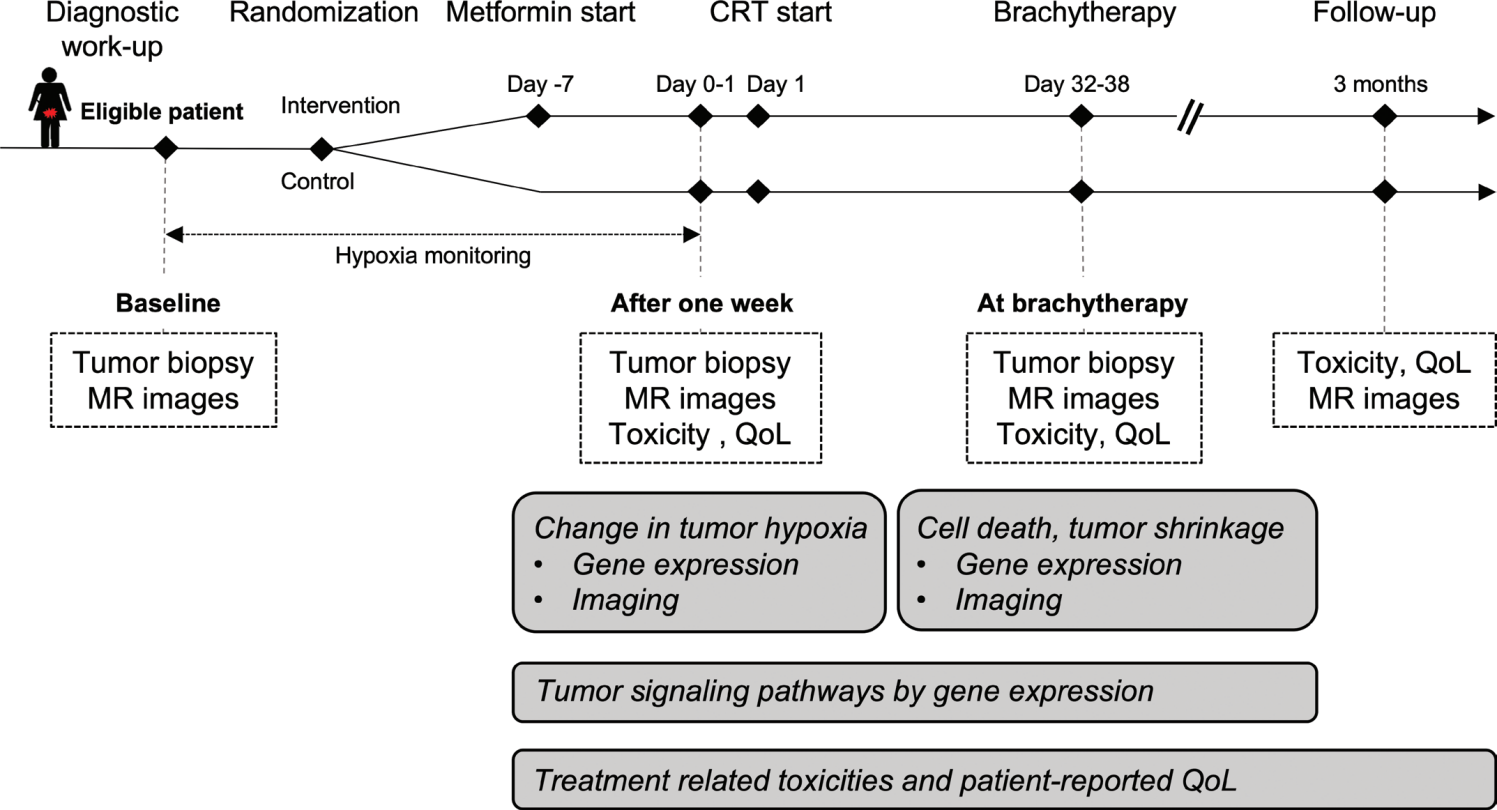
Schematic overview of the Metoxy-lacc study. CRT: chemoradiotherapy.

Included patients were allocated with a 1:1 ratio to receive standard chemoradiotherapy with or without metformin (Figure 1). A simple randomization procedure was performed, and a predefined, computer-generated allocation sequence was applied. Standard chemoradiotherapy included external beam radiotherapy (EBRT) of 45 Gray (Gy) to the pelvis ± paraaortic region, and a simultaneous integrated boost of 2.2–2.3 Gy to pathological lymph nodes. This was followed by a high dose rate, MRI-guided, adaptive brachytherapy boost, aiming to achieve a minimal total dose to 90% of the high-risk clinical target volume (HR-CTV) between 85 and 95 Gy in 2 Gy equivalent doses. Concurrent cisplatin (40 mg/m^2^), or carboplatin if contraindicated, was administered weekly during EBRT. Patients randomized to intervention received peroral metformin 1 week prior to the start of chemoradiotherapy with a flat dosage of 850 mg twice per day, and the treatment was continued throughout chemoradiotherapy.

Patients were scheduled for a study specific follow-up 3 months after completed chemoradiotherapy. Albeit not a study-specific procedure, a pelvic MRI was performed in accordance with standard institutional practice.

### Hypoxia biomarkers

To assess the imaging- and gene-based hypoxia biomarkers, pelvic MR images and tumor biopsies were collected at inclusion (baseline), after 1 week immediately before the start of chemoradiotherapy, and at the first fraction of brachytherapy (Figure 1). MRI at baseline and at brachytherapy were already included in the clinical routine.

MRI at baseline and after 1 week was performed at the same Siemens MAGNETOM Vida fit 3T, and included T_1_-weighted (T_1_W) and T_2_-weighted (T_2_W), diffusion-weighted (DW) and dynamic contrast enhanced (DCE) images (Supplementary Table 1). MRI after 1 week had a modified protocol compared to the baseline MRI, which included omission of certain imaging sequences (coronal and sagittal diffusion-weighted images, high resolution post-contrast T_1_W images and T_2_W oblique images). The modified protocol significantly reduced image acquisition time without affecting biomarker construction.

MRI at brachytherapy included T2W- and DW- images (Supplementary Table 1).

The MRI-based biomarker was constructed using the oxygen consumption and supply hypoxia (CSH) imaging method, where the apparent diffusion coefficient (ADC) and K^trans^ were extracted from DW- and DCE-images, respectively, and combined voxel-wise into hypoxia images [20, 22]. Hypoxic fraction was calculated for each tumor and time point, and the same weighting factor between ADC and K^trans^ as in the previous work was applied [20, 23]. To ensure that no increase in hypoxia occurred prior to the start of chemoradiotherapy, hypoxia images at baseline and after 1 week were consecutively compared in each patient by personnel blinded for treatment allocation.

Biopsy sampling at baseline and at the first fraction of brachytherapy was performed under anesthetics along with other planned standard procedures, while study-specific biopsies after 1 week were acquired without anesthetics. The gene-based biomarker was planned to be constructed from the expression level of six hypoxia-related genes, as previously described [4]. A total of 1–4 biopsies, approximately 5×5 mm in size, were collected from each tumor, and immediately snap frozen and stored at –80°C for the upcoming analysis.

### Toxicity

Physician-reported acute toxicity was assessed by Common Terminology Criteria for Adverse Events (CTCAE) version 4.0. For the continuous evaluation of safety, reporting of adverse events (AEs) was limited to CTCAE ≥ Grade 3 and serious adverse events (SAEs) due to the expected high frequency of acute toxicity associated with standard chemoradiotherapy. For renal toxicity, all AEs, regardless of CTCAE grade, were reported due to the increased risk of lactic acidosis associated with renal failure. Metformin was consistently held if creatinine clearance dropped below 60 ml/min.

Planned assessment of all physician-reported acute toxicity was performed at baseline, after 4 weeks of chemoradiotherapy, at the end of treatment and after 3 months of follow-up (Figure 1). Further, patient-reported Quality of Life (QoL) was assessed by the European Organization for Research and Treatment of Cancer Quality of Life (EORTC-QLQ) Core 30 (C30), Cervix 24 (CX24) and Endometrial 24 (EN24) questionnaires at baseline, during treatment and after 3 months of follow-up (Figure 1).

### Study hypothesis and endpoints

The main study hypothesis was that metformin, applied 1 week prior to chemoradiotherapy, could decrease tumor hypoxia at clinically tolerable doses. Further, our MRI- and gene-based hypoxia biomarkers were hypothesized to be clinically feasible and hence, implementable in the routine clinical workflow.

Primary endpoint was change in the gene-based hypoxia biomarker from baseline to 1 week prior to the start of chemoradiotherapy (Figure 1). Secondary endpoints were changes in functional MRI parameters (hypoxia, ADC, K^trans^) after 1 week, tumor shrinkage at the first fraction of brachytherapy and acute toxicity (Figure 1).

Exploratory endpoints were change in patient-reported quality of life from baseline, disease free and overall survival, and change in tumor signaling pathways assessed by global gene expression data.

Tolerability, safety and feasibility were not protocol-defined endpoints, but are reported here as they were considered important methodological aspects.

### Statistical methods

A Student *t*-test was applied to calculate sample size for the primary endpoint. Initial calculations were based on modest assumptions regarding metformin effect in patient tumors, since no data were available in the literature. Initially, the study sample size of 90 patients was powered to detect a change in the gene-based biomarker of 0.34 [24], with a standard deviation (SD) of 0.7, 5% significance level and 90% confidence level.

Patient accrual rate was slower than expected, mainly due to the covid pandemic and competing studies. When results from a similar metformin study in LACC patients were published [16], the sample size was redetermined with updated estimates for expected change and SD in the gene-based biomarker, and with a confidence level of 80%. This yielded a final sample size of 42 patients.

**Figure 2:**
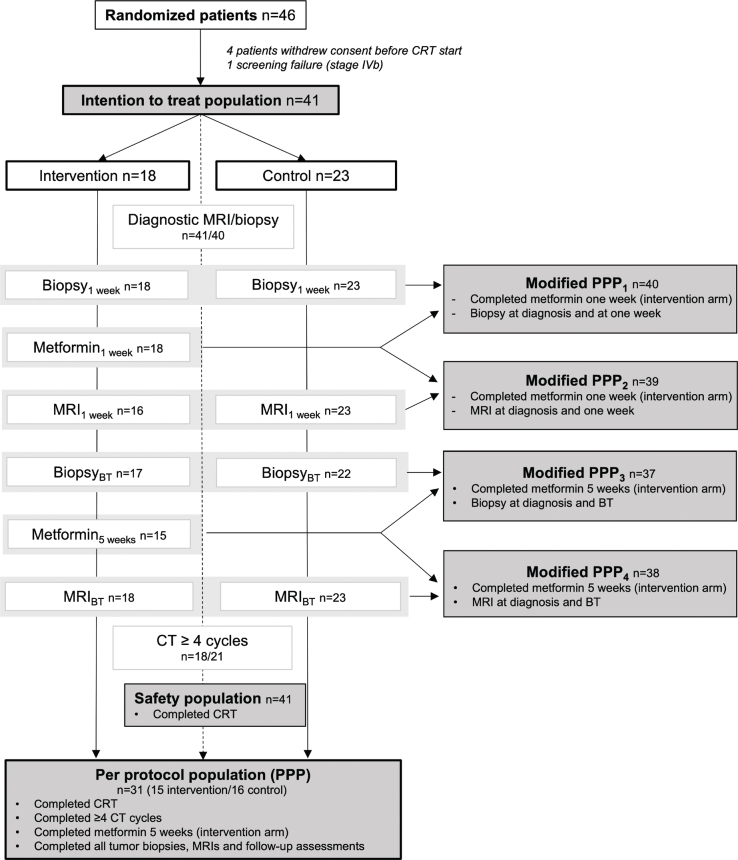
Overview of patient populations for analyses. Population criteria and number of patients (*n*) are indicated. Primary endpoint will be assessed in the modified PPP_1_, while secondary endpoints will be assessed in the modified PPP_2_, modified PPP_3_ and safety population. CRT: chemoradiotherapy; BT: brachytherapy; CT: chemotherapy; PPP: per protocol population.

Tolerability and safety were reported here as treatment completion rates (population for analyses, Figure 2) and the proportion of patients with reported AEs grade ≥ 3 and SAEs, respectively, and compared between arms. Feasibility was reported as the proportion of patients in the respective populations for analyses relative to the intention to treat (ITT) population (Figure 2). Fisher’s exact test and Student’s unpaired *T*-test were used to determine differences between groups.

### Ethics, registration and funding

The study was conducted in accordance with the World Medical Association Declaration of Helsinki, Council for International Organizations of Medical Sciences (CIOMS) International Ethical Guidelines, ICH Good Clinical Practice (GCP) Guidelines and General Data Protection Regulation (GDPR). The study was approved by the Regional Ethics Committee for Medical and Health Research in Southeast Norway, the Norwegian Medical Products Agency and the data protection office at Oslo University Hospital. All patients provided signed informed consent. The study was registered at ClinicalTrials.gov with identifier NCT04275713.

## Results

At study closure, 46 patients were randomized. Two patients in each arm withdrew consent before the start of chemoradiotherapy. In the intervention arm, the two patients withdrew after 0 and 3 days of metformin treatment. One patient was excluded due to stage IVb disease (screening failure). Thus, 41 patients were available for analyses, which included 18 and 23 patients in the intervention and control arm, respectively (Figure 2).

There were no significant differences in baseline clinical and treatment characteristics between study arms (Table 1). Most patients had squamous cell carcinoma and stage T2b disease, and were non-smokers.

**Table 1:** Clinical and treatment characteristics.

	Intervention (*n* = 18)	Control (*n* = 23)	*P* *
**Age**			0.45
Median (range)	56 (36–73)	49 (31–72)	
**Smoking**			0.68
Yes	2	4	
No	15	19	
NA	1	-	
**FIGO_2018_-stage**			0.83
Ib2-3	2 (11%)	3 (13%)	
II	9 (50%)	9 (39%)	
III–IVa	7 (39%)	11 (48%)	
**TNM-stage**			0.37
T1b2-3N0-2M0	2 (11%)	3 (13%)	
T2N0-2M0	14 (78%)	20 (87%)	
T3-4aN0-2M0	2 (11%)	0	
**Tumor size (GTV-T)** (cm^3^)			0.32
Mean (range)	26 (3–90)	20 (2–58)	
**Histology**			0.40**
Squamous cell carcinoma	12 (67%)	17 (74%)	
Adenocarcinoma	4 (22%)	6 (26%)	
Adenosquamous carcinoma	1 (5.5%)	0	
Poorly diff. carcinoma	1 (5.5%)	0	
**Pathological lymph nodes**			
Pelvic	6 (33%)	11 (48%)	0.52
Paraaortic	0	0	
**Hemoglobin level** (g/dl)			0.95
Mean (range)	13 (10–14)	13 (10–14)	
**Chemotherapy cycles**			0.50
0–1 cycles	0	0	
2–3 cycles	0	2	
4–6 cycles	18	21	
**Total dose HR-CTV D90** (Gy)			0.98
Mean (range)	92 (89–94)	92 (86–94)	
**Treatment days**			0.16
Mean (range)	42 (38–49)	44 (38–49)	
**Metformin treatment**			-
Dose (mg)			
Mean (range)	68,189 (12,750–88,400)	-	
Days			
Mean (range)	40 (7.5–52)	-	

* *P*-values from student’s *T*-test and Fisher’s exact test are indicated.

** Analysis performed only with squamous cell carcinoma and adenocarcinoma.

NA: not available.

All patients completed chemoradiotherapy within 50 days and with a total dose to the HR-CTV within recommended values. In total, 39 patients (all patients in the intervention arm and 91% in the control arm) completed ≥4 chemotherapy cycles. Two patients, one in each arm, received carboplatin due to baseline contradictions. All patients in the intervention arm completed the first week of metformin, while 15 patients completed a total of 5 weeks. Metformin was temporarily held in two patients and discontinued in three patients due to AEs. In the latter three patients, metformin was discontinued in one patient due to allergic urticaria, while the other two patients preferred to discontinue metformin due to lower grade gastrointestinal toxicity.

Biopsies applicable for biomarker assessment were missing for one patient at baseline and two patients at the time of brachytherapy. All patients had a biopsy at 1 week, leaving proportions for completed biopsy procedures of 98, 100 and 95%, at the three consecutive timepoints, respectively. There were no patient complications related to the biopsy sampling procedures.

All scheduled MRI examinations were completed, but the images were of unacceptable quality for biomarker assessment in two patients. All follow-up visits were completed in all patients.

The final per protocol population (PPP), defined as patients completing EBRT, brachytherapy, ≥4 cycles of concurrent chemotherapy, all biopsies and MRIs and all follow-up assessments, included 31 patients (76% of ITT population, Figure 2). The completion rate was higher in the intervention arm (83%) compared to the control arm (70%) (Figure 2). Forty patients (98% of ITT population) were available for analyses of the primary endpoint (modified PPP_1_, Figure 2).

**Figure 3:**
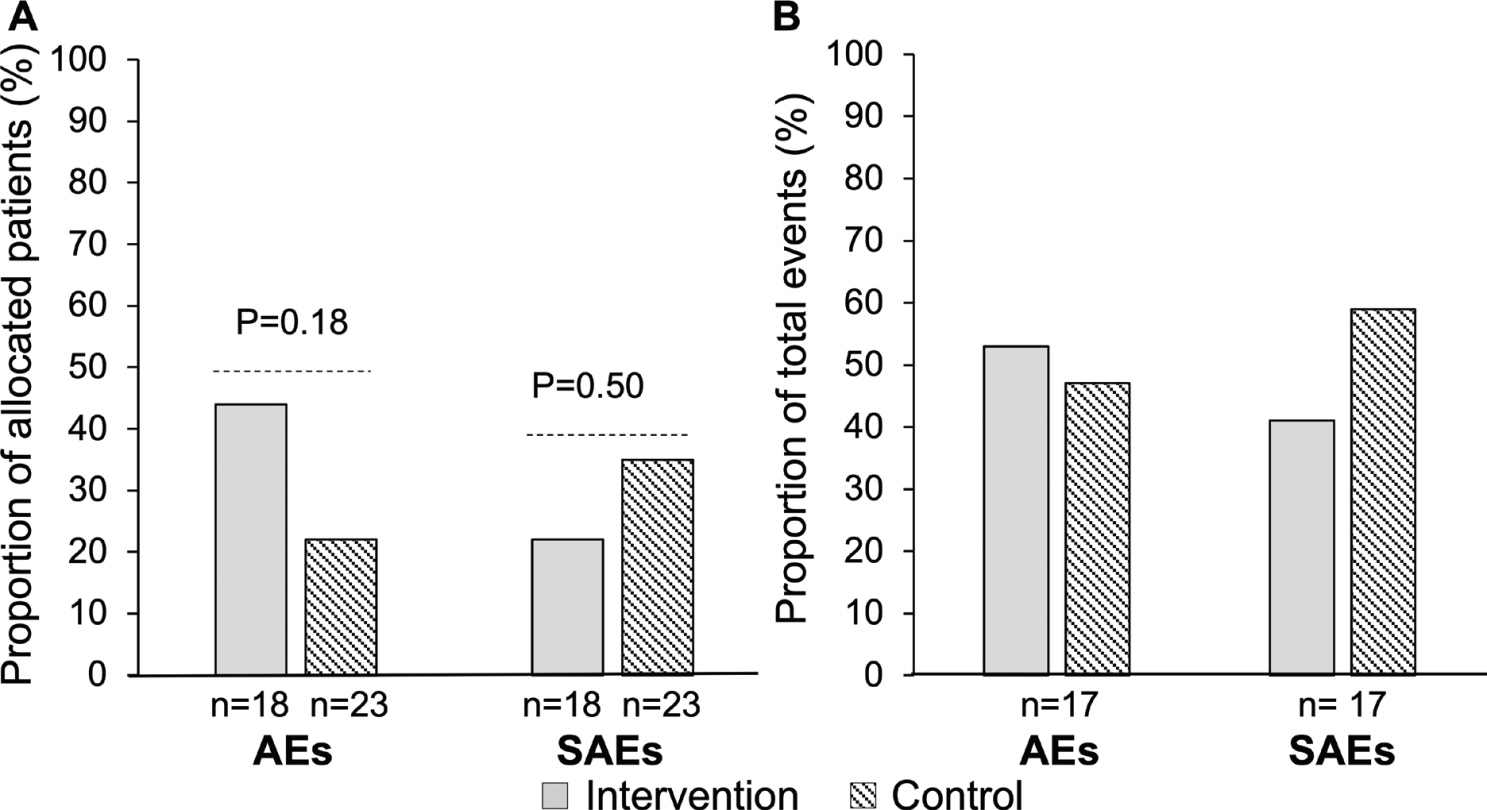
Distribution of AEs (CTCAE Grade ≥3) and SAEs between study arms. (A) presents proportions of patients in each arm with AEs and SAE, while (B) presents proportions of the total number of AEs (CTCAE Grade ≥3) and SAEs in each arm (B). *P*-values from Fisher’s exact test are indicated. AE: adverse events; SAE: serious adverse events; CTCAE: Common Terminology Criteria for Adverse Events.

In total, 17 AEs and 17 SAEs were reported in 13 and 12 patients, respectively. The proportions of patients with AEs ≥grade 3 and SAEs in each arm were not statistically different between the two study arms (Figure 3A). In total 53% of AEs and 41% of SAEs occurred in the intervention arm (Figure 3B). Most AEs were gastrointestinal toxicity. Renal toxicity occurred in one patient in the intervention arm (CTCAE grade 1). All SAEs were related to hospital admissions for management of toxicities.

**Figure 4:**
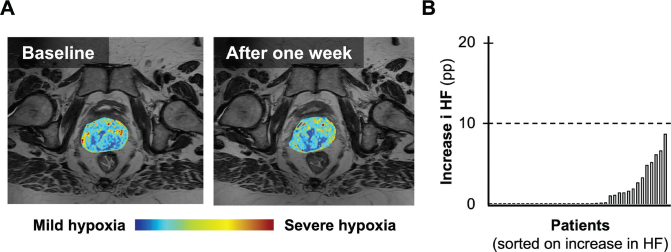
Example of hypoxia images at baseline and after 1 week for one study patient (A). The hypoxia images are overlaid on a T2W-MR image. A bar chart presenting patients with an increase in imaging-based hypoxic fraction (HF) after 1 week when compared to baseline (B). The increase is presented as percentage points (pp). Patients without increase in hypoxic fraction are presented with an increase of zero. The patients are presented in incremental order with respect to increase in hypoxic fraction, irrespective of inclusion number. The dotted line represents the safety limit of 10 pp.

Hypoxia monitoring by MR-imaging, blinded for treatment allocation, did not show any increase in hypoxia that gave rise to safety concerns regarding metformin effect (Figure 4). In some tumors there was a slight increase in hypoxic fraction. These increases ranged from 0.1 to 8.7 percentage points (pp) with a mean of 2.9 pp, and were all below 10 pp, which was considered a reasonable safety limit. Premature closure of the study due to a possible metformin-induced increase in hypoxic fraction was therefore not needed.

## Discussion

The main objective of this sub study was to investigate whether metformin in combination with chemoradiotherapy in LACC patients was tolerable and safe. Further, we aimed to determine whether our imaging- and gene-based hypoxia biomarkers were feasible in a prospective, interventional trial. Study procedures were adapted to the clinical workflow to enhance feasibility and exploit the existing information already collected as part of routine cervical cancer staging and treatment.

All patients completed planned chemoradiotherapy. The two patients who received a suboptimal chemotherapy dose (<4 cycles) were both in the control arm. The majority of patients completed metformin for 5 weeks, and a higher proportion of patients in the intervention arm completed the allocated treatment and study-specific procedures. There was no significant difference in the proportion of patients experiencing AEs ≥ grade 3 and SAEs between the study arms. Moreover, evaluation of hypoxia images after 1 week revealed no increase in hypoxia above the safety limit. Metformin combined with chemoradiotherapy thereby presents as a tolerable and safe intervention, and thereby overcomes an important barrier for clinical implementation.

The survival benefit of hypoxic modification is likely limited to patients with clinically significant hypoxia [25], which demonstrates the need to include feasible methods for hypoxia assessment when interventional efficacy shall be determined [17]. In LACC, MR imaging is a state-of-the-art clinical routine both before and during treatment, and these images may be exploited for hypoxia evaluation without imposing extra costs or patient distress [26]. The imaging parameters K^trans^ and ADC, which are required for construction of hypoxia images, have been shown to be both reproducible and reliable [27, 28]. Moreover, the high spatial resolution of MR images ensures a precise definition of tumor areas [29]. The repeated application of gadolinium-based contrast agent was administered within current recommendations [30]. Our MRI-based hypoxia biomarker thereby presents as a feasible and appealing approach for the evaluation of efficacy in individual patients.

Hypoxia PET tracers such as 18F-fluoroazomycin arabinoside (^18^F-FAZA) and 18F-fluoromisonidazole (^18^F-FMISO) are increasingly applied for hypoxia assessment in clinical hypoxia targeting studies [16, 31, 32]. These imaging techniques are expensive, often unavailable, and impose additional patient burden, and are therefore less applicable in clinical practice [33]. Furthermore, hypoxia PET images suffer from low reproducibility, and reliable imaging is particularly challenging in cervical tumors where tracer accumulation in the bladder may result in an overestimation of the hypoxic volume [34, 35].

Our MRI-based hypoxia biomarker is well-suited for continuous evaluation of treatment response to ensure efficacy in individual patients [17, 21, 36]. Monitoring of potential treatment induced changes in individual tumors is particularly relevant when introducing experimental drugs with uncertain biological effect. This was clearly demonstrated in a clinical study on LACC patients by Milosevic et al., where response monitoring by DCE-MRI revealed an increase, rather than the intended decrease, in tumor hypoxia after 7 days of sorafenib treatment [21]. This finding led to a premature closure of the study, to avoid exposure of additional patients to a possibly harmful intervention [21, 36]. In contrast, we found no increase in hypoxia above the safety limit in our study population. The addition of metformin therefore presents as a safe intervention in patients with LACC.

The study design, where metformin is administered 1 week before chemoradiotherapy, allows for enhanced insight into molecular pathways affected by metformin through gene expression analyses of tumor biopsies. Further, the synchronized imaging and biopsy sampling allows for repeated biological validation of the imaging-based hypoxia biomarker [19]. Cervical tumors are easily accessible by clinical examinations, and as our study demonstrates, repeated tumor biopsies are feasible in LACC patients.

In our study, we aimed to evaluate clinical implementation of our hypoxia biomarkers. Almost all cervical tumors contain some hypoxic areas [23, 37], and a metformin effect was expected even in tumors with a small hypoxic fraction. Hence, patients were included in the study irrespective of hypoxia status. However, in a study designed to assess survival benefit, patient selection based on clinically relevant hypoxia would be more appropriate. Metformin is a low cost and globally available drug. This facilitates conduction of larger multicenter studies that are powered to determine effects on disease control and survival. Thus, although our study is the largest interventional study investigating metformin in LACC, the number of included patients was limited and only powered to evaluate whether metformin could decrease hypoxia in LACC patients, according to our biomarkers.

The application of hypoxia biomarkers for patient classification and treatment selection is challenged by the current lack of reproducible thresholds that reflects clinically relevant hypoxia, referring to the extent of hypoxia with impact on patient outcome [18]. Hence, further validation of our MRI- and gene-based biomarker would enhance clinical relevance. The widespread availability of MRI facilitates the acquisition of sufficient evidence for determination of applicable thresholds.

In conclusion, we have demonstrated that metformin in combination with chemoradiotherapy is tolerable and safe, and that our imaging- and gene-based hypoxia biomarker approach is feasible in a prospective study. Results are awaited regarding metformin efficacy in hypoxia modification.

## Supplementary Material

Tolerability, safety and feasibility of metformin combined with chemoradiotherapy in patients with locally advanced cervical cancer: a phase II, randomized study

## Data Availability

The imaging and gene data are not publicly available due to Data Protection Regulations.
