# Supplementary material for: Tolerability, safety and feasibility of metformin combined with chemoradiotherapy in patients with locally advanced cervical cancer: a phase II, randomized study
Source: Acta Oncol. 2025 Mar 19;64:43045. doi: 10.2340/1651-226X.2025.43045 (PMC11971942; doi:10.2340/1651-226X.2025.43045)
Supplement: Tolerability, safety and feasibility of metformin combined with chemoradiotherapy in patients with locally advanced cervical cancer: a phase II, randomized study [file AO-64-43045-s1.pdf]

Supplementary material has been published as submitted. It has not been copyedited, or typeset by Acta Oncologica

Supplementary Table 1.

MR image acquisition parameters

	Baseline	One week
Magnetic field strength	3T	3T
Overall acquisition time (min)	45	25
**T2W-MRI**		
Sequence type	T2W 3D SPACE	T2W 3D SPACE
Echo time (ms)	96	96
Repetition time (ms)	1200	1200
In-plane resolution (mm^3)	0.45 x 0.45	0.45 x 0.45
Slice thickness (mm)	1	1
**DW-MRI**		
Sequence type	Transversal 2D Spin Echo ZOOMit	Transversal 2D Spin Echo ZOOMit
b-values (s/mm^2)	0,200,1000	0,200,1000
Slice thickness (mm)	4	4
Slice gap (mm)	1	1
FOV (mm^2)	160 x 152	160 x 152
Repetition time (ms)	4300	4300
Echo time (ms)	64	64
Total acquisition time (s)	210	210
Number of averages	12	12
In-plane resolution (mm^2)	1 x 1	1 x 1
**DCE-MRI**		
Sequence type	3D T1W spoiled gradient recalled echo (spgr) Dixon with water reconstruction	3D T1W spoiled gradient recalled echo (spgr) Dixon with water reconstruction
Slice thickness (mm)	3.3	3.3
FOV (mm^2)	210 x 210	210 x 210
Matrix	320 x 320 x 48	320 x 320 x 48
Repetition time (ms)	4.39	4.39
Echo time (ms)	1.3	1.3
Temporal resolution (s)	12	12
In-plane resolution (mm^2)	0.66 x 0.66	0.66 x 0.66
